# Comparative analysis of four nutritional scores predicting the incidence of MACE in older adults with acute coronary syndromes after PCI

**DOI:** 10.1038/s41598-023-47793-3

**Published:** 2023-11-21

**Authors:** Xing-Yu Zhu, Dan-Dan Yang, Kai-Jie Zhang, Hui-Jing Zhu, Fei-Fei Su, Jian-Wei Tian

**Affiliations:** 1 Graduate School of Hebei North University, Zhangjiakou, 075031 Hebei China; 2 Xuzhou Central Hospital, General Practice Medicine, Xuzhou, 221009 Jiangsu China; 3 Department of Cardiovascular Medicine, Air Force Characteristic Medical Center, Beijing, 100142 China

**Keywords:** Interventional cardiology, Nutrition

## Abstract

To determine the most appropriate nutritional assessment tool for predicting the occurrence of major adverse cardiovascular events (MACE) within 1 year in elderly ACS patients undergoing PCI from four nutritional assessment tools including PNI, GNRI, CONUT, and BMI. Consecutive cases diagnosed with acute coronary syndrome (ACS) and underwent percutaneous coronary intervention (PCI) in the Department of Cardiovascular Medicine of the Air force characteristic medical center from 1 January 2020 to 1 April 2022 were retrospectively collected. The basic clinical characteristics and relevant test and examination indexes were collected uniformly, and the cases were divided into the MACE group (174 cases) and the non-MACE group (372 cases) according to whether a major adverse cardiovascular event (MACE) had occurred within 1 year. Predictive models were constructed to assess the nutritional status of patients with the Prognostic Nutritional Index (PNI), Geriatric Nutritional Risk Index (GNRI), Controlling nutritional status (CONUT) scores, and Body Mass Index (BMI), respectively, and to analyze their relationship with prognosis. The incremental value of the four nutritional assessment tools in predicting risk was compared using the Integrated Discriminant Improvement (IDI) and the net reclassification improvement (NRI). The predictive effect of each model on the occurrence of major adverse cardiovascular events (MACE) within 1 year in elderly ACS patients undergoing PCI was assessed using area under the ROC curve (AUC), calibration curves, decision analysis curves, and clinical impact curves; comparative analyses were performed. Among the four nutritional assessment tools, the area under the curve (AUC) was significantly higher for the PNI (AUC: 0.798, 95%CI 0.755–0.840 P < 0.001) and GNRI (AUC: 0.760, 95%CI 0.715–0.804 P < 0.001) than for the CONUT (AUC: 0.719,95%CI 0.673–0.765 P < 0.001) and BMI (AUC: 0.576, 95%CI 0.522–0.630 P < 0.001). The positive predictive value (PPV) of PNI: 67.67% was better than GNRI, CONUT, and BMI, and the negative predictive value (NPV): of 83.90% was better than CONUT and BMI and similar to the NPV of GNRI. The PNI, GNRI, and CONUT were compared with BMI, respectively. The PNI had the most significant improvement in the Integrated Discriminant Improvement Index (IDI) (IDI: 0.1732, P < 0.001); the PNI also had the most significant improvement in the Net Reclassification Index (NRI) (NRI: 0.8185, P < 0.001). In addition, of the four nutritional assessment tools used in this study, the PNI was more appropriate for predicting the occurrence of major adverse cardiovascular events (MACE) within 1 year in elderly ACS patients undergoing PCI.

## Introduction

As the global population continues to age, the over-65s and over-80s will be the fastest-growing segments of the population^1^. As a result, the elderly population will continue to grow and there will be a further increase in the number of elderly patients with acute coronary syndromes. Acute coronary syndrome (ACS) is an acute ischemic syndrome caused by rupture of unstable atherosclerotic plaques or fresh thrombosis secondary to erosion in the coronary artery^2^. ACS is an important cause of disability and death in patients^3^. With the rapid development of percutaneous coronary intervention (PCI), PCI has become the mainstay of treatment for patients with ACS^4,5^.Despite medical advances such as PCI, ACS still has high mortality, and MACE rates^6,7^. Along with the increasing number of elderly ACS patients treated with PCI, the regression after PCI has also become a widespread concern. Recent evidence suggests that malnutrition is an important factor in the prognosis of cardiovascular (CV) disease^8^. However, the nutritional status of patients is often overlooked, despite its association with poor outcomes in patients with cardiovascular disease^9,10^. Nutritional status affects patient regression after surgery, so early identification of patient nutritional status facilitates clinicians to make early clinical decisions and interventions to optimize clinical management to improve patient prognosis^11^.

The Prognostic Nutritional Index (PNI), Geriatric Nutritional Risk Index (GNRI), Controlling Nutritional Status (CONUT) score, and Body Mass Index (BMI) can be quickly calculated based on blood parameters and height and weight and can indicate the nutritional status of the patient simply and objectively, which is widely used in patients with cardio-cerebral vascular diseases^12,13^. The PNI is a nutritional assessment tool based on serum albumin levels and lymphocyte counts; it was initially used to assess immunological and nutritional aspects in patients undergoing gastrointestinal surgery^14^. It has since been used for a wide range of other diseases, such as cancer, chronic kidney disease, and cardiovascular disease^15^. There is evidence that all of these factors are associated with the prognosis of patients with acute coronary syndrome (ACS)^4^. The prognostic value of low albumin in ACS and stable coronary artery disease (including previous myocardial infarction and heart failure) has been reported^16^. Another study concluded that low albumin in stable coronary artery disease (CAD) is caused by atherosclerotic systemic inflammation^17^. However, the GNRI, which takes into account both serum albumin levels and body weight, is commonly used to assess the nutritional status of hospitalized older adults^18^. Underweight elderly patients with ACS also have a higher risk of MACE in the year following PCI than normal-weight patients. BMI is an indirect indicator of body fat based on height and weight and has traditionally been used for nutritional assessment in adult men and women^19^. Bucholz et al.^20^ suggested that low BMI is associated with increased short- and long-term mortality after AMI. The CONUT score was developed by Ulibarri et al.^21^ in 2005 as a screening tool for nutritional status in hospitalized patients. It is a composite indicator based on total lymphocyte count, total cholesterol, and serum albumin proposed to assess the nutritional status of patients. However, to date, it remains elusive which score is more effective in predicting the incidence of MACE in elderly ACS patients undergoing PCI. Therefore, we used four objective nutritional status assessment tools to predict the prognosis of elderly patients undergoing PCI for ACS and explored which nutritional assessment tool is more suitable for nutritional assessment of elderly patients undergoing PCI for ACS, to provide clinicians with a reference for early clinical decision-making and intervention.

## Methods

### Study design and selection of patients

**Figure 1:**
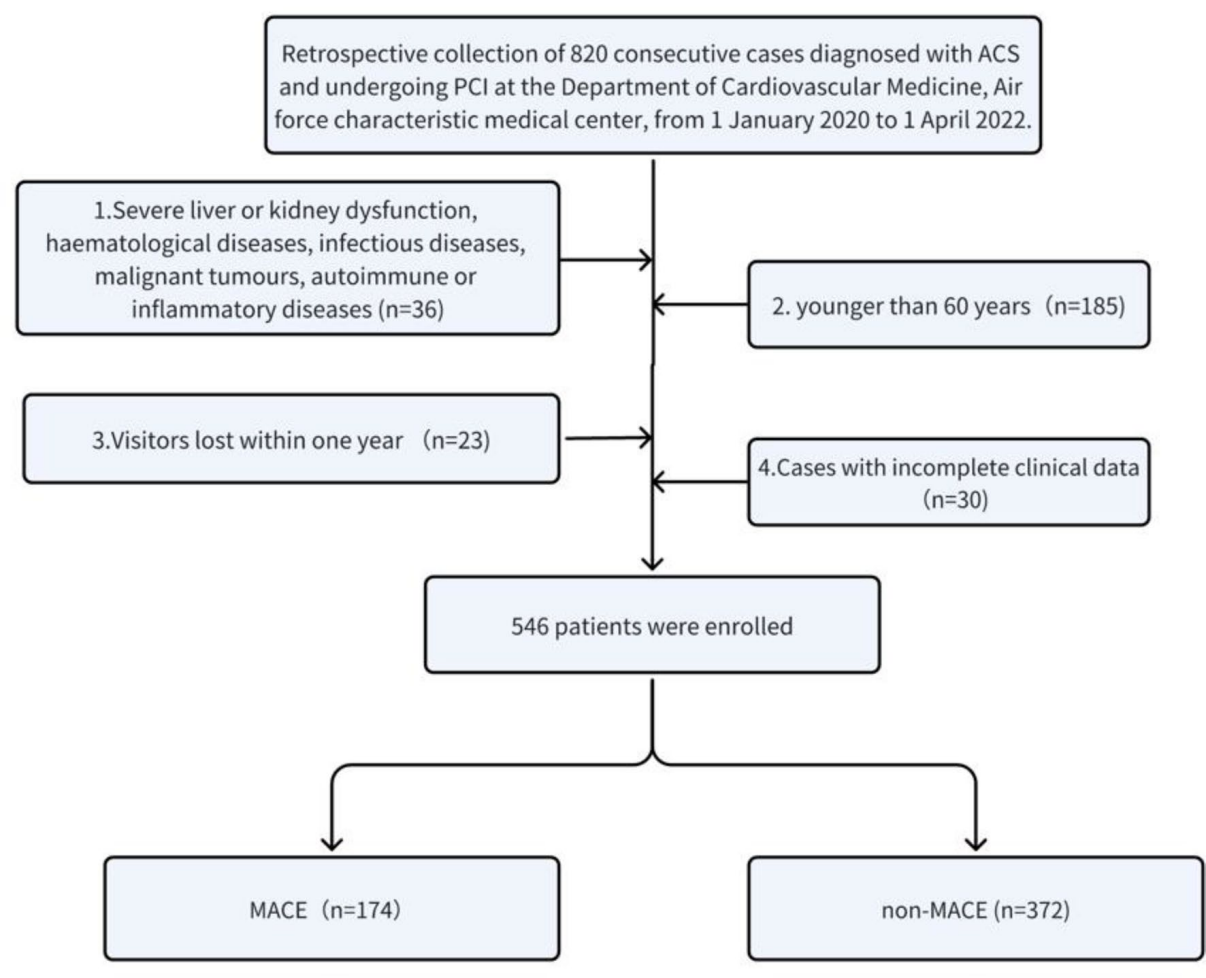
Flow diagram indicating study population.

This study was approved by the Ethics Committee of the Air force characteristic medical center. All our research methods are by the Helsinki Declaration and relevant guidelines/regulations and all participants informed consent to the study. Informed consent was obtained from all participants in the study. In this retrospective, observational, single-center study, we retrospectively collected 618 consecutive cases diagnosed with acute coronary syndrome (ACS) and underwent percutaneous coronary intervention (PCI) in the Department of Cardiovascular Medicine at the Air Force Specialty Medical Center from 1 January 2020 to 11 April 2022, and 546 cases were finally included according to the inclusion and exclusion criteria, of which 426 were male 120 females, aged 60–95 years. They were followed up for 1 year and divided into MACE group (174 cases) and non-MACE group (372 cases) according to the occurrence of major adverse cardiovascular events (MACE) within 1 year (Fig. 1).

### Inclusion criteria

(1) age ≥ 60 years; (2) patients with ACS treated with PCI; refer to “Acute coronary syndromes” for diagnostic criteria of ACS^2^; (3) MACE criteria: included recurrent angina, restenosis, cardiac death, acute myocardial infarction, and hospital readmission for cardiovascular reasons (unstable angina/severe arrhythmia/heart failure, etc.); and (4) complete clinical case information.

### Exclusion criteria

(1) Insufficient relevant case information; (2) Patients with a combination of severe hepatic/renal dysfunction, hematological disease, infectious disease, malignancy, autoimmune or inflammatory disease. Patients with one or more of the above conditions are excluded.

### Methods

Basic data collection ① Patient's basic clinical characteristics: collect the patient's age, gender, height, weight, admission blood pressure, resting heart rate, smoking history, and drinking history. ② The patient's previous medical history: history of hyperlipidemia, hypertension, diabetes mellitus, peripheral vascular disease, cerebrovascular disease, and so on. ③ Auxiliary examination: coronary angiography: location of diseased coronary vessels, degree of stenosis of diseased vessels, number of diseased vessel branches, etc. Cardiac ultrasound: left ventricular ejection fraction (LVEF). Blood routine (white blood cells, neutrophils, lymphocytes, hemoglobin, platelets, ultrasensitive C-reactive protein); coagulation function, liver function, renal function, cardiac infarction three, blood lipids, electrolytes, and so on.

### Telephone follow-up

The follow-up endpoint event was the occurrence of a major adverse cardiovascular event within 1 year; follow-up began on 1 January 2020 and ended on 31 April 2023; the occurrence of a major adverse event after the patient's discharge was recorded.

### Nutritional status assessment tools

Prognostic Nutritional Index (PNI), Geriatric Nutritional Risk Index (GNRI), Controlling Nutritional Status (CONUT) score, and Body Mass Index (BMI). The formulae for PNI, GNRI, and BMI were based on previous literature and were as follows: PNI = albumin (g/L) + 5 × lymphocytes (× 10^9^/L); GNRI = [1.489 × serum albumin (g/L)] + [41.7 × (body weight (kg)/ideal body weight (kg)); and BMI = body weight (kg)/height (cm)^2^; The CONUT score was developed by Ulibarri et al.^21^ in 2005 as a screening tool for nutritional status of hospitalized patients. It is a composite index based on total lymphocyte count, total cholesterol, and serum albumin proposed to assess the nutritional status of patients.

### Statistical analysis

Statistical analyses were performed using R (version 4.2.3) and SPSS(version 25.0) software, normally distributed measures were expressed as mean ± standard deviation ($\overline{x}$ ± *s*) and comparisons were made using a t-test; Non-normally distributed measures were expressed as median (M(Q1, Q3)) and comparisons were made using the Mann–Whitney U test; counts were expressed as percentages using the χ^2^ test; and analyses were performed using Spearman's correlation analysis. To assess the utility of each nutritional status assessment tool on the occurrence of adverse cardiovascular events within 1 year after PCI in elderly ACS patients, we plotted the area under the curve (AUC) of subjects' work characteristics (ROC) to assess the sensitivity and specificity of the four nutritional status assessment tools on the occurrence of MACE within 1 year after PCI in elderly ACS patients. The difference was considered statistically significant at P < 0.05. We calculated the net reclassification index (NRI) using the 'survival' and 'nricens' packages in the R software and the integrated discriminant improvement index (IDI) using the 'PredictABEL', 'survival' and 'rms' packages to assess the incremental value of different dietary assessment tools. Associations between dietary scores and outcomes were examined using one-way logistic regression analyses. Using the occurrence of MACE in elderly ACS patients 1 year after PCI as the dependent variable and four dietary assessment tools including PNI, GNRI, CONUT and BMI as the independent variables, a Nomogram prediction model was constructed using the ‘survival’, ‘rms’ and ‘nomogramFormula’ packages in R software. We used the 'regplot' and 'rmda' packages in the R software to construct calibration curves, clinical decision curves and clinical impact curves to assess the discrimination, calibration and clinical impact of the prediction models.

## Results

### Baseline characteristics

A total of 618 consecutive cases diagnosed with acute coronary syndrome (ACS) and undergoing percutaneous coronary intervention (PCI) at the Department of Cardiovascular Medicine, Air force characteristic medical center between 1 January 2020 and 11 April 2022 were collected, and 546 cases were finally included according to the inclusion and exclusion criteria. In 546 patients, the incidence of MACE within 1 year was 31.9%. The comparison of the proportions of males, hypertension, peripheral vascular disease, cerebrovascular disease, diabetes mellitus, and platelet count between the 2 groups showed no statistically significant difference (P > 0.05). The MACE group had significantly higher proportions of age, smoking, heart rate, hyperlipidemia, leukocyte count, neutrophils, high-sensitivity C-reactive protein, creatinine, uric acid, and myoglobin than the non-MACE group, Creatine kinase isoenzyme, troponin, LDL, B-type natriuretic peptide, CONUT score, and GRACE score were significantly higher than those in the non-MACE group, and systolic blood pressure, lymphocytes, hemoglobin, albumin, body mass index, LVEF, PNI and GNRI were significantly lower than those in the non-MACE group, with statistically significant differences (P < 0.05, Table 1).

**Table 1:** Comparison of general clinical data between MACE and non-MACE groups.

Characteristic	Non-MACE (n = 372)	MACE (n = 174)	*P* value
Age [years old, M (Q1, Q3)]	65.00 (62.00, 70.00)	69.00 (65.00, 77.00)	0.001
Gender (male, %)	290 (78.0)	136 (78.2)	0.957
Smoking, n (%)	119 (32.0)	93 (53.4)	0.001
Heart rate [n/min, M (Q1,Q3)]	72.00 (67.00, 80.00)	77.00 (68.00, 90.00)	0.001
Past history n (%)			
Hyperlipidemia	138 (37.1)	84 (48.3)	0.013
High blood pressure	253 (68.0)	122 (70.1)	0.621
Peripheral vascular disease	26 (7.0)	8 (4.6)	0.281
Cerebral vascular disease	61 (16.4)	34 (19.5)	0.367
Diabetes	140 (37.6)	79 (45.4)	0.084
Systolic blood pressure (mmHg, ${\overline{\text{x}}}$ ± *s*)	133.00 (121.00, 143.00)	122.50 (113.50, 137.00)	0.001
White blood cell count [×10^9^/L,M(Q1,Q3)]	6.70 (5.60, 8.23)	7.50 (6.16, 10.80)	0.001
Neutral particle count [×10^9^/L,M(Q1,Q3)]	4.21 (3.30, 5.29)	6.30 (4.79, 8.99)	0.001
Lymphocyte [×10^9^/L,M(Q1,Q3)]	1.63 (1.28, 2.02)	1.25 (0.97, 1.71)	0.001
Hemoglobin [g/L,M(Q1,Q3)]	142.00 (134.00, 152.00)	136.00 (116.50, 147.50)	0.001
Platelet count [×10^9^/L,M(Q1,Q3)]	210.00 (176.00, 241.00)	220.50 (178.00, 245.00)	0.223
Hs-CRP[mg/L,M(Q1,Q3)]	1.20 (0.50, 5.10)	3.74 (0.67, 30.59)	0.001
Creatinine [μmol/L,M(Q1,Q3)]	74.00 (65.00, 84.00)	88.00 (72.50, 108.50)	0.001
Uric acid [μmol/L,M(Q1,Q3)]	341.00 (286.50, 389.50)	357.50 (294.00, 426.00)	0.022
Albumen [g/L,M(Q1,Q3)]	44.20 (42.80, 46.00)	40.20 (38.60, 41.80)	0.001
Myoglobin [μg/L,M(Q1,Q3)]	56.00 (36.00, 74.50)	82.50 (54.50, 263.45)	0.001
Creatine kinase isoenzyme [μg/L,M(Q1,Q3)]	2.00 (2.00, 3.35)	4.35 (2.00, 40.70)	0.003
Troponin [μg/L,M(Q1,Q3)]	0.01 (0.01, 0.91)	0.13 (0.01, 5.25)	0.001
TC [mmol/L,M(Q1,Q3)]	3.99 (3.34, 5.24)	3.80 (3.17, 4.25)	0.001
TG [mmol/L,M(Q1,Q3)]	1.39 (1.06, 2.03)	1.31 (1.02, 1.72)	0.047
HDL [mmol/L,M(Q1,Q3)]	1.03 (0.91, 1.20)	1.00 (0.85, 1.15)	0.042
LDL-C [mmol/L,M(Q1,Q3)]	2.04 (1.57, 2.62)	2.49 (2.02, 2.90)	0.001
NT-proBNP [ng/L,M(Q1,Q3)]	35.90 (17.75, 88.90)	153.45 (56.60, 378.80)	0.001
BMI (kg/m^2^, ${\overline{\text{x}}}$ ± *s*)	25.71 (24.03, 27.73)	25.05 (22.49, 27.41)	0.004
LVEF [%,M(Q1,Q3)]	61.00 (58.00, 64.00)	56.00 (51.50, 59.00)	0.001
PNI [M(Q1,Q3)]	52.70 (49.75, 55.80)	46.35 (43.45, 49.72)	0.001
GNRI [M(Q1,Q3)]	115.60 (110.53, 119.68)	107.49 (101.10, 112.56)	0.001
CONUT [M(Q1,Q3)]	3.00 (3.00, 4.00)	4.00 (3.00, 5.00)	0.001
GRACE score [M(Q1,Q3)]	94.00 (83.00, 106.00)	113.50 (99.50, 126.00)	0.001

MACEs: major adverse cardiovascular events; Hs-CRP: hypersensitive C-reactive protein; TC : serum total cholesterol; TG: triglyceride; HDL-C: high-density lipoprotein-cholesterol; LDL-C: low-density lipoprotein-cholesterol; NT-proBNP: N-terminal pro-B-type natriuretic peptide; LVEF: left ventricular ejection fraction; PNI: Prognostic Nutritional Index ;GNRI: Geriatric Nutritional Risk Index; CONUT: controlling nutritional status ;BMI: Body Mass Index; GRACE: global registry of acute coronary events.

### ROC curves for MACE

**Figure 2:**
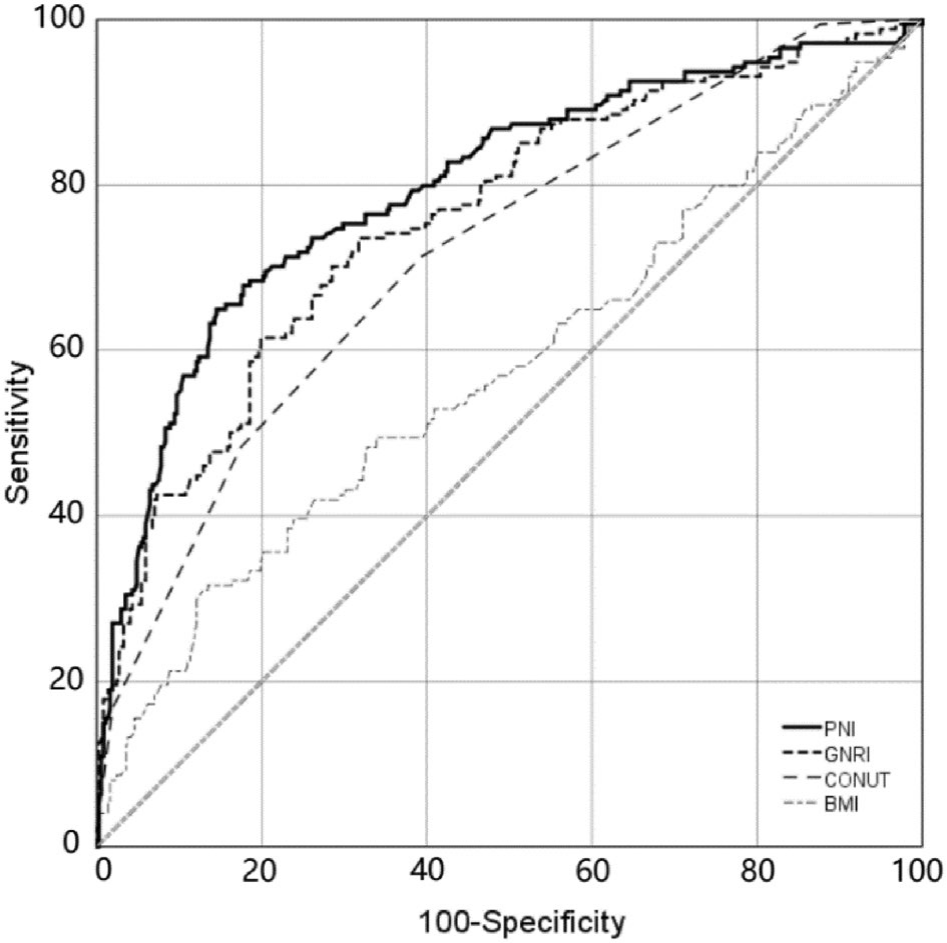
PNI, GNRI, CONUT and BMI predict ROC of MACE within 1 year after PCI in elderly patients with ACS. ACS Acute Coronary Syndromes, PCI Percutaneous Coronary Intervention, MACE Major Adverse Cardiovascular Event, PNI Prognostic Nutritional Index, GNRI Geriatric Nutritional Risk Index, CONUT Controlling nutritional status, BMI Body Mass Index, ROC Receiver Operating Characteristic.

In this study, ROC curves were analyzed for PNI, GNRI, CONUT, and BMI for the prediction model of MACE within 1 year in elderly ACS patients who underwent PCI (Fig. 2 and Table 2).In terms of AUC, the area under the curve (AUC) was significantly higher for PNI (AUC: 0.798, 95%CI 0.755–0.840 P < 0.001) and GNRI (AUC: 0.760, 95%CI 0.715–0.804 P < 0.001) than for CONUT (AUC:0.719, 95%CI 0.673–0.765 P < 0.001) and BMI (AUC:0.576, 95%CI 0.522–0.630 P < 0.001). The established Jordon's index was used to determine the cut-off values for PNI, GNRI, CONUT, and BMI, respectively; and to calculate the sensitivity, specificity, positive predictive value (PPV), and negative predictive value (NPV), respectively (Table 2). PPV of PNI: 67.67% was better than GNRI, CONUT, and BMI, and NPV: 83.90% was better than CONUT and BMI and similar to NPV of GNRI. (Table 2).

**Table 2:** Comparison of AUCs, PPV, and NPV of nutritional assessment tools.

Models	AUC	95%CI	*P* value for AUCs	Cut-off value	Sensitivity	Specificity	PPV	NPV	*P* value
PNI	0.798	0.755–0.840	< 0.001	47.98	0.649	0.855	67.67%	83.90%	< 0.001
GNRI	0.760	0.715–0.804	< 0.001	112.33	0.736	0.683	52.03%	84.67%	< 0.001
CONUT	0.719	0.673–0.765	< 0.001	3.50	0.713	0.608	45.93%	81.88%	< 0.001
BMI	0.576	0.522–0.630	0.004	23.00	0.316	0.866	48.14%	70.97%	0.001

PPV Positive Predictive Value, NPV Negative Predictive Value, AUC Area Under the Curve. CI Confidence Intervals.

In addition, to assess the incremental value of the Nutrition Assessment Tool (NAT) in predicting the occurrence of MACE within 1 year, we analyzed the four indicators using the Integrated Discriminant Improvement Index (IDI) and the Net Reclassification Index (NRI). The accuracy of the predictive model can be assessed through the net reclassification index (NRI). If the NRI exceeds 0, it indicates that the new model is superior to the old one, while a negative value indicates the opposite. The calculation of the Integrated Discrimination Improvement (IDI) was based on the predicted probabilities of the disease models for each individual. The IDI indicates the change in the gap between the two models' forecasts. Overall, it is suggested that the greater the IDI, the stronger the predictive ability of the new model. If IDI > 0, it is an improvement; if IDI < 0, it is a negative improvement; if IDI = 0, it is no improvement in the new model. The PNI, GNRI, and CONUT were used to compare with BMI, respectively. The most significant improvement in IDI was found for PNI (IDI: 0.1732, p < 0.001); the most significant improvement in NRI was also found for PNI (NRI: 0.8185, p < 0.001) (Table 3).

**Table 3:** Comparison of IDI and NRI for PNI, GNRI, CONUT and BMI.

Models	IDI			NRI		
	Absolute IDI	95% CI	*P* value	Total NRI	95% CI	*P* value
PNI	0.1732	0.1402–0.2062	< 0.001	0.8185	0.6342–1.0146	< 0.001
GNRI	0.126	0.0982–0.1537	< 0.001	0.7879	0.4716–0.9745	< 0.001
CONUT	0.1041	0.0756–0.1327	< 0.001	0.5471	0.3420–0.7215	< 0.001

IDI, Integrated Discrimination Improvement; NRI, Net Reclassification Improvement.

### Single-factor logistic regression analysis

With the occurrence of MACE in elderly ACS patients 1 year after PCI as the dependent variable, and four nutritional assessment tools, including PNI, GNRI, CONUT, and BMI, as the independent variables, one-way logistic regression analyses were used to calculate the ratio of ratio (OR) and 95% confidence intervals (CI). If the regression coefficient in the regression analysis is positive and the OR is greater than 1, the factor is determined to be a risk factor for the outcome; conversely, it is a protective factor and an OR value of 1 means that the factor does not play a role in the occurrence of the disease. The results showed that the p-values of the four dietary assessment tools were less than 0.05, proving that the four independent variables were statistically significant and all had independent influences. The incidence of MACE increased by 0.804% for every 1 decrease in PNI, by 0.881% for every 1 decrease in GNRI, by 2.145% for every 1 increase in CONUT score, and by 0.91% for every 1 decrease in BMI (Table 4). High PNI, high GNRI and high BMI were protective factors and high CONUT was a risk factor (Table 4).

**Table 4:** Single-factor logistic regression analysis.

Models	β	SE	OR	95%*CI*	*P* value
PNI	-0.219	0.023	0.804	0.768–0.841	< 0.001
GNRI	-0.127	0.014	0.881	0.857–0.906	< 0.001
CONUT	0.763	0.094	2.145	1.785–2.579	< 0.001
BMI	-0.094	0.030	0.910	0.858–0.965	0.002

β: regression coefficient; SE: Standard Error; OR: Odds Ratios, Indicates the ratio of the exposure ratio of a factor in the case group to the exposure ratio of that factor in the control group, which may reflect the fact that the proportion of a factor in the case group is several times higher than that in the control group. In the present study, it was possible to analyse the associations between the four dietary assessment tools and the presence or absence of MACE in elderly ACS patients 1 year after PCI.

### Predictive modeling and evaluation

**Figure 3:**
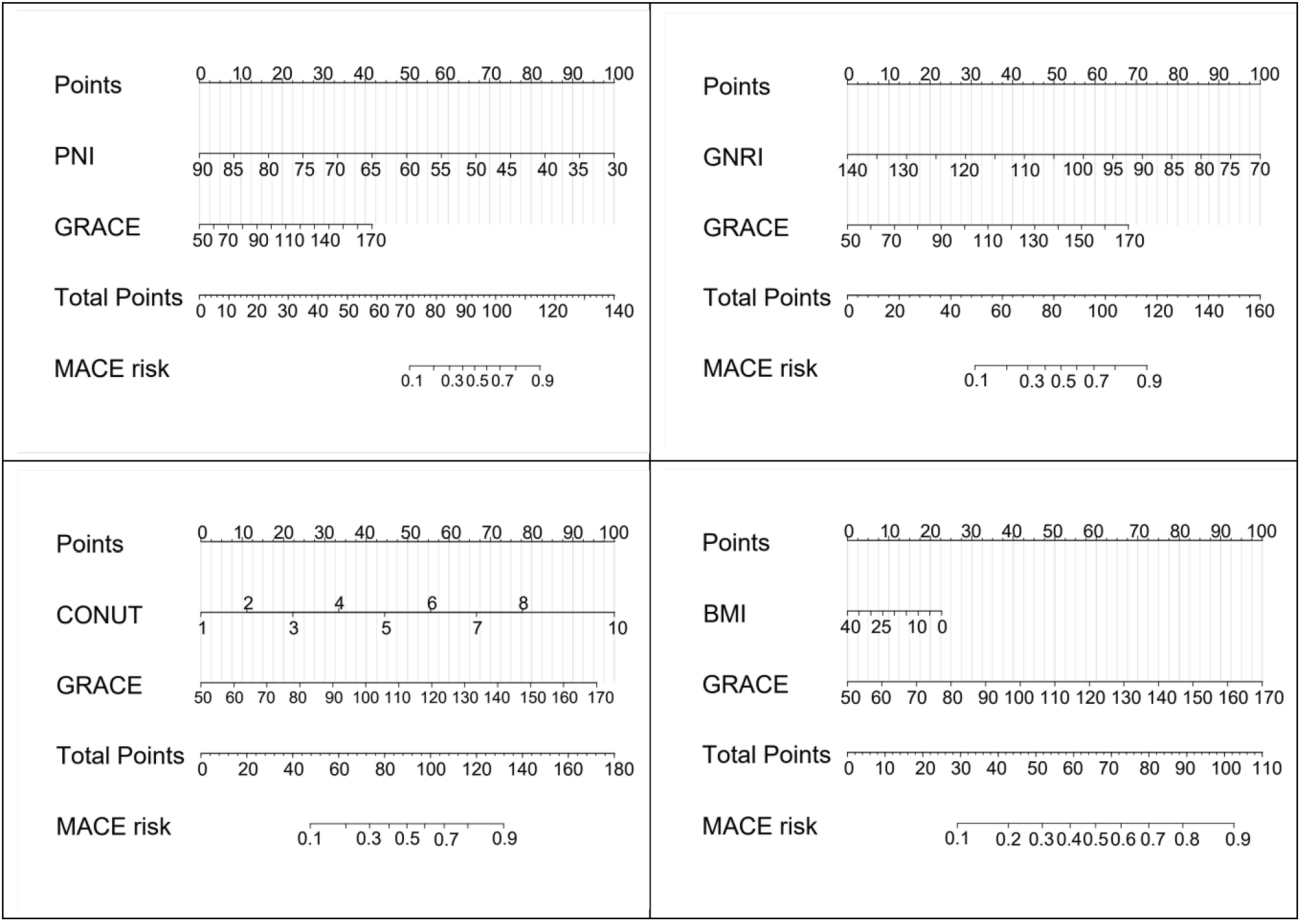
Clinical predictive model nomogram for the incidence of MACE at 1 year after PCI in elderly ACS patients.

**Figure 4:**
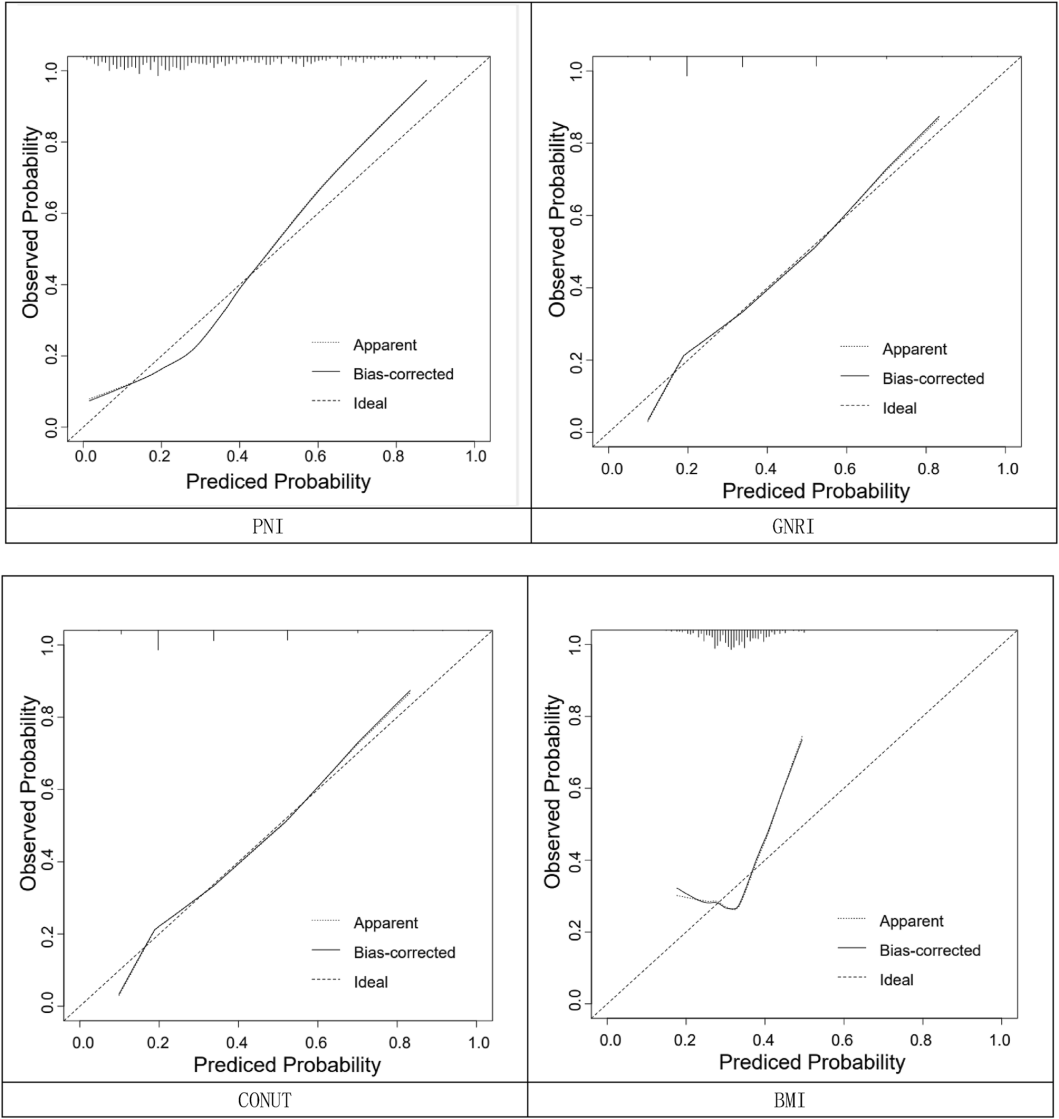
Calibration curve of the clinical prediction model for the occurrence of MACE 1 year after PCI in elderly patients with ACS. Calibration curves indicate the goodness-of-fit of the nomogram. The 45° straight line represents the perfect match between the actual (Y-axis) and nomogram-predicted (X-axis) probabilities. A closer distance between two curves indicates higher accuracy.

**Figure 5:**
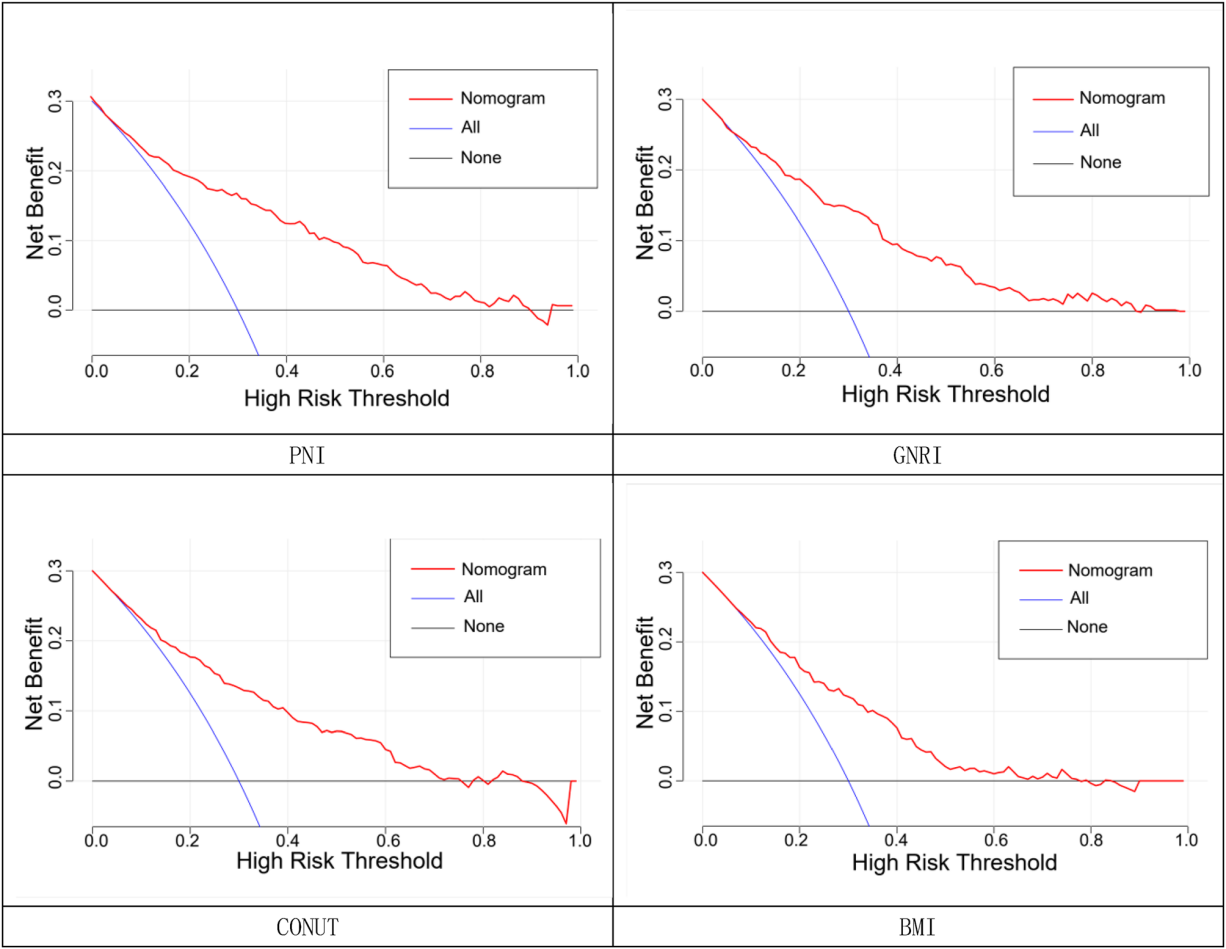
Decision curve analysis of a clinical prediction model for the occurrence of MACE 1 year after PCI in elderly ACS patients. Decision curve analysis evaluates the net benefit of a model or test in comparison to the two default strategies of treat all patients and treat no patients. The y-axis indicates the net benefit; x-axis indicates threshold probability. In the figure above, there is a black line, a blue line and a red line. The black line means that all people are not treated, then the net benefit of treatment must be 0. The blue line means that all people are treated, then the value decreases as the threshold probability increases. The red line is a line plot of threshold probability versus net benefit for the decision model. Using the black and blue lines as a reference, a model with a red line close to the reference line indicates that there is no application value, and a model above the reference line within a large threshold interval indicates a better model. As can be seen in the figure, the model constructed using PNI has a better net benefit in the threshold probability interval of 0.15 to 0.8 and a higher net benefit than the other models.

**Figure 6:**
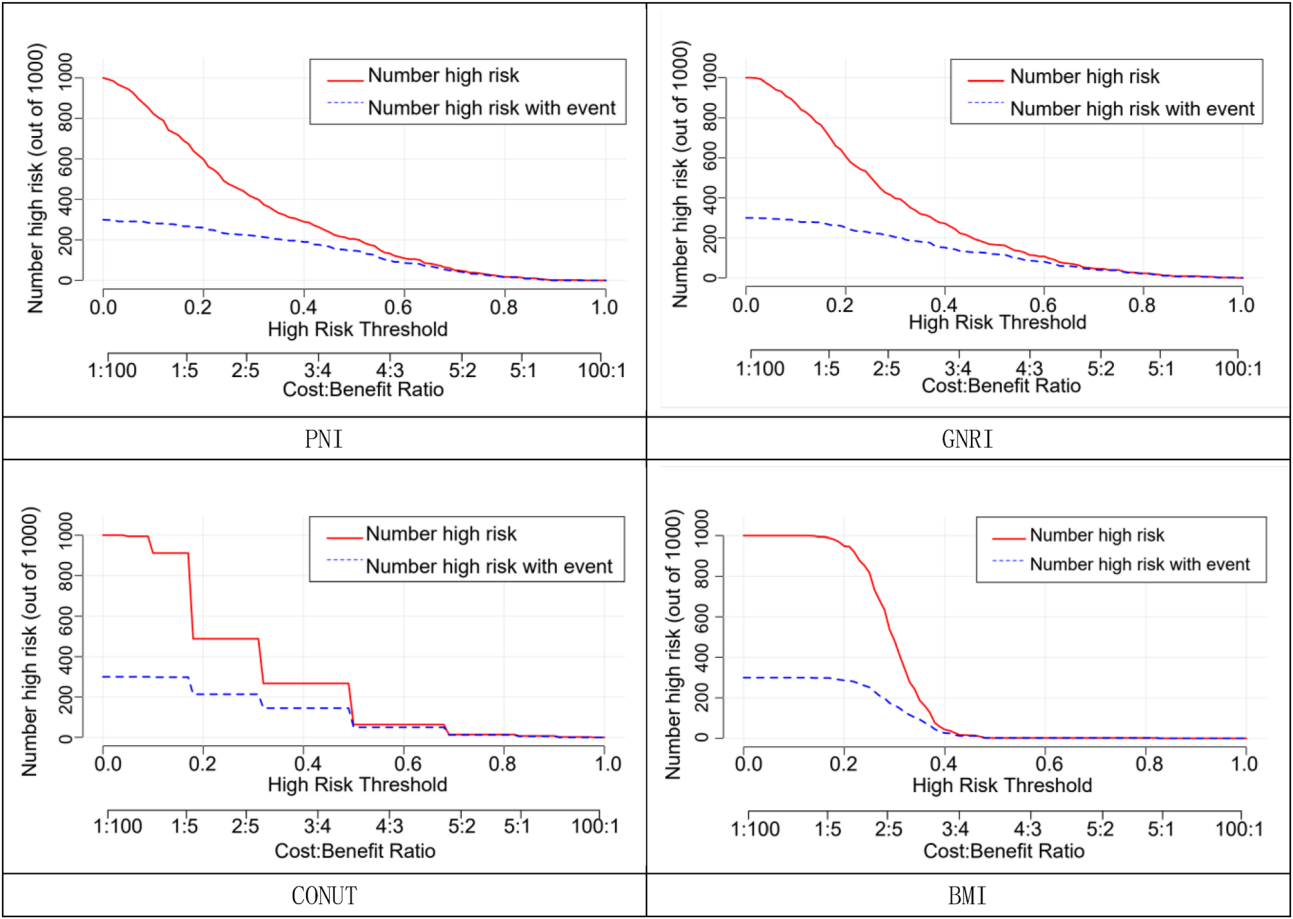
Clinical impact curves of a clinical prediction model for the occurrence of MACE 1 year after PCI in elderly ACS patients. The y-axis indicates the number high risk (out of 1000); x-axis indicates threshold probability and benefit ratio. The red line is the number of people at high risk at different thresholds, the blue dotted line is the number of people who actually had a positive outcome occur at different thresholds, and the number of misdiagnoses between the red and blue lines.

Four nutritional assessment tools, including PNI, GNRI, CONUT and body mass index, were each used to construct a clinical prediction model nomogram for the incidence of MACE at 1 year after PCI in elderly ACS patients (Fig. 3); A more intuitive understanding of MACE incidence can be obtained based on the total score in the graph. According to the calibration curves, the three prediction models constructed by PNI, GNRI, and CONUT showed good calibration ability (Fig. 4); Decision-analysis curves (DCA) suggested that PNI and GNRI at threshold probabilities greater than 15% and CONUT at threshold probabilities greater than 20% were more favourable for predicting the risk of MACE 1 year after PCI in elderly ACS patients using this prediction model than implementing an intervention programme for all patients, with the net benefit of the prediction model being significantly higher than that of all or no intervention. Predictive models constructed from BMI, on the other hand, have poor clinical validity (Fig. 5). Clinical impact curves (CICs) were further plotted based on DCA to assess the clinical impact of each model, showing the estimated number of people predicted to have MACE and the actual number of people with the disease at each risk threshold; the PNI and GNRI constructed models had a lower rate of misdiagnosis than the CONUT and BMI constructed models (Fig. 6).

## Discussion

This study aimed to identify the most appropriate nutritional assessment tool for predicting the occurrence of major adverse cardiovascular events (MACE) within 1 year in elderly ACS patients undergoing PCI from four nutritional assessment tools including PNI, GNRI, CONUT, and BMI. Currently, the commonly used nutritional assessment tools are divided into subjective nutritional assessment tools and objective nutritional assessment tools. Subjective nutritional assessment tools are more limited in their application to this group of patients, whereas the four nutritional assessment tools, such as PNI, GNRI, CONUT, and BMI, can be quickly assessed by only requiring the results of routine blood tests in the clinic and height and weight^22–24^. Previous studies have shown that malnutrition is common in patients with chronic heart failure, valvular disease, or coronary artery disease and is associated with a poor prognosis^4,25,26^. Yuan et al.^27^ used PNI, GNRI, and CONUT to define malnutrition, which was associated with a significantly increased risk of death in old age. To our knowledge, no studies are comparing PNI, GNRI, CONUT, and BMI in ACS patients undergoing PCI. Assessing the nutritional status of elderly patients with ACS has been recognised as difficult due to time constraints in the acute care setting and other potential risk factors. Therefore, it is imperative to find a nutritional assessment tool that is more suitable for assessing the occurrence of major adverse cardiovascular events (MACE) within 1 year in elderly ACS patients undergoing PCI.

A recent study by Tonet et al.^28^ found that almost 44% of elderly ACS patients were malnourished or at risk of malnutrition. This illustrates the prevalence of malnutrition in ACS patients. However, clinical cardiologists are unaware of the prevalence of malnutrition in older patients with ACS. Malnutrition often goes unnoticed and untreated despite its growing importance. The four nutritional assessment tools analysed in this study are easy to calculate. They can be used to identify patients at risk of malnutrition. However, a possible explanation for malnutrition in older ACS patients who have undergone PCI is that nutritional status may be a surrogate indicator of inflammation^29^. Chronic inflammatory diseases predispose to lower albumin levels^30^. High levels of malnutrition are associated with high levels of inflammation, and these factors contribute to an increased atherosclerotic burden. The link between these three is known as the malnutrition-inflammation-atherosclerosis syndrome^31^. Therefore, to reduce the risk of MACE in patients, it is also important to control inflammation. The studies by Li et al.^32,33^ provide potential clues to the development of more rational disease control strategies for our continued research. ODE-based theoretical modeling studies on gene/protein signaling networks have been equally important for the study of understanding regulatory mechanisms and finding potential therapeutic targets in diseases. In order to better predict the malnutrition problem, it is also very important to start with some basic research. Xu et al.^34^ proposed that the specificity and competitiveness of mRNAs are dominant in protein phase separation, which is a good direction for us to study. We need to focus not only on clinical research but also on basic research to understand the nature of the disease.

In this study, nutritional status was assessed by PNI, GNRI, CONUT, and BMI, and its association with the occurrence of major adverse cardiovascular events (MACE) within 1 year in elderly patients undergoing PCI for ACS was evaluated. The main results were as follows: among the four assessment tools, the AUC, PPV, and NPV of PNI and GNRI were significantly higher than those of CONUT and BMI; From the AUC, it was found that PNI and GNRI predicted the MACE risk rate better. From the positive and negative predictive values, PNI and GNRI were found to be more likely to predict non-MACE; this finding is consistent with the results of other studies^25,35^. From the Integrated Discriminant Improvement Index (IDI) and the Net Reclassification Index (NRI), the PNI predicted the greatest incremental value of risk, and patients with low nutrition scores had a higher risk of MACE than those with high nutrition scores^36^.

Clinical Prediction Models, also referred to as Clinical Prediction Rules, Prognosis Models, or Risk Scores, are models comprising multiple risk factors that calculate the likelihood of developing a disease or the occurrence of a particular outcome in the future^37^. One type of modeling is prognostic modeling, which centers on assessing the likelihood of potential outcomes such as disease recurrence, mortality, incapacity, or complications manifesting at some point in the future based on the patient's current state of health^38^. Clinical prediction models can evaluate and classify the risk of patients based on fundamental clinical features and tests and examinations, thus aiding the identification of medium- and high-risk individuals early on. This can help clinicians create sensible management strategies and measures for controlling risk factors for patients outside of the hospital setting. In addition, the study of interaction prediction in various fields of computational biology provides valuable research directions. The study by Sun et al.^39^ provided a new deep learning algorithm called Graph Convolutional Networks with Graph Attention Networks (GCNAT), which promises to be a useful biomedical research tool for predicting potential metabolite disease associations in the future. It may be more convenient to analyse the nutritional status of ACS patients and allow us to better predict the risk of MACE in elderly ACS patients. Wang et al.^40^ developed a novel deep learning prediction model called DMFGAM that could become a powerful tool for predicting hERG channel blockers in the early stages of drug discovery and development. Predictive modelling will be widely used in the future to help clinicians make better diagnoses and treatments.

We constructed a column-line diagram of the clinical prediction model for the occurrence of MACE within 1 year after PCI in elderly ACS patients with each of the four dietary assessment tools. The risk of developing MACE can be suggested more intuitively. According to the calibration curves, the three prediction models constructed from PNI, GNRI, and CONUT had good calibration ability; the decision analysis curves (DCA) suggested that the models constructed from PNI and GNRI had high clinical validity, while the prediction model constructed from BMI had poor clinical validity. The Clinical Impact Curve (CIC) suggests that the PNI and GNRI constructs have a lower rate of misdiagnosis than the CONUT and BMI constructs. The PNI can better predict the risk of developing MACE, as reflected more intuitively by the predictive model we constructed.

There are many screening tools for malnutrition, but there is no consensus on which screening tool to use in patients with ACS. Based on our results, we suggest using the PNI score, which uses only 2 laboratory values and is very easy to calculate even without a specific automated calculator. Screening for malnutrition in elderly ACS patients undergoing PCI may identify patients at high risk of adverse cardiovascular outcomes who may benefit from targeted secondary prevention programs with supplementation to improve their prognosis.

## Limitations of the study

The present study is a single-centre retrospective study with a relatively small number of patients and therefore has some drawbacks. There is no information in this study about patients' economic status, education, adherence, etc., which might help us to understand the causal factors of malnutrition. We did not compare the prognostic value of a nutritional screening tool with a more sophisticated comprehensive nutritional assessment. This is because malnutrition is a complex problem, especially in the elderly, with diverse etiologies and a wide range of determinants. The validity of assessing nutritional status through simple screening tools (PNI, GNRI, CONUT, and BMI) alone remains uncertain because of the lack of comparison with comprehensive nutritional assessments, such as subjective holistic assessments and mini-nutritional assessments. We only assessed nutritional status on admission and did not examine the relationship between changes in nutritional status over time and the incidence of MACE after PCI in elderly ACS patients. The results still need further validation with large samples and multicenter data. We welcome additions and improvements to this study from other researchers and medical centres in different countries.

## Data Availability

The datasets used and/or analysed during the current study are available from the corresponding author on reasonable request.

## Abbreviations

ACS: Acute coronary syndromes
PCI: Percutaneous coronary intervention
MACE: Major adverse cardiovascular event
PNI: Prognostic Nutritional Index
GNRI: Geriatric Nutritional Risk Index
CONUT: Controlling nutritional status
BMI: Body Mass Index
ROC: Receiver operating characteristic
AUC: Area under the curve
IDI: Integrated discrimination improvement
NRI: Net reclassification improvement
PPV: Positive predictive value
NPV: Negative predictive value
OR: Odds ratios
CI: Confidence intervals
SE: Standard error
GRACE: Global registry of acute coronary events
